# EGFR-TKIs - induced cardiotoxicity in NSCLC: incidence, evaluation, and monitoring

**DOI:** 10.3389/fonc.2024.1426796

**Published:** 2024-06-24

**Authors:** Yunlong Wang, Qinggui Qiu, Xuan Deng, Mengchao Wan

**Affiliations:** ^1^ Department of Oncology, Jiangxi Provincial People’s Hospital, The First Affiliated Hospital of Nanchang Medical College, Nanchang, Jiangxi, China; ^2^ Department of Outpatient, Jiangxi Provincial People’s Hospital, The First Affiliated Hospital of Nanchang Medical College, Nanchang, Jiangxi, China

**Keywords:** non-small cell lung cancer, targeted drugs, cardiotoxicity, epidermal growth factor receptor inhibitors, evaluation and monitoring

## Abstract

The advent of targeted drug therapy has greatly changed the treatment landscape of advanced non-small cell lung cancer(NSCLC), but the cardioxic side effects of targeted drug anti-cancer therapy seriously affect the prognosis of NSCLC, and it has become the second leading cause of death in cancer patients. Therefore, early identification of the cardiotoxic side effects of targeted drugs is crucial for the prevention and treatment of cardiovascular diseases. The cardiotoxic side effects that may be caused by novel targeted drugs epidermal growth factor receptor inhibitors, including thromboembolic events, heart failure, cardiomyopathy, arrhythmia and hypertension, are discussed, and the mechanisms of their respective adverse cardiovascular reactions are summarized, to provide useful recommendations for cardiac management of patients with advanced lung cancer to maximize treatment outcomes for lung cancer survivors. Clinicians need to balance the risk-benefit ratio between targeted therapy for malignant tumors and drug-induced cardiotoxicity, and evaluate and monitor TKIs-induced cardiotoxicity through electrocardiogram, cardiac imaging, biomarkers, etc., so as to remove the susceptibility risk factors as soon as possible and provide a reference for the clinical use of such drugs in the treatment of malignant tumors.

## 1 Introduction

Whether in the world or in China, the incidence rate and mortality of primary bronchogenic lung cancer (hereinafter referred to as lung cancer) rank first among all malignant tumors (1). In 2022, it is estimated that there will be about 870,000 cases and 760,000 deaths of lung cancer in China (2), and non-small cell lung cancer (NSCLC) is the most common type of lung cancer, accounting for 80% of lung cancers (3). Most patients are at an advanced stage at the time of diagnosis, and miss the best time for surgical treatment, which significantly affects the prognosis of patients with advanced lung cancer, making chemotherapy become the traditional standard of care for advanced NSCLC (4). However, the plateau phase of chemotherapy response and its adverse effects limit its clinical use. Molecularly targeted therapy has become the first-line treatment for advanced NSCLC due to its efficacy, specificity, and low adverse reactions (5). Clear-cut therapeutic targets for NSCLC include epidermal growth factor receptor (EGFR), anaplastic lymphoma kinase, mesenchymal epidermal conversion factor, and human epidermal growth factor receptor (HER), as well as vascular endothelial growth factor, monoclonal antibodies, and multi-targeted small molecule inhibitors.

The aberrant expression of EGFR is closely related to the invasion and metastasis of tumor cells, tumor angiogenesis, chemotherapy resistance, and abnormal cell proliferation. Overexpression and mutations of EGFR have been found in patients with NSCLC (6), therefore, tumor cell proliferation can be effectively inhibited by counteracting EGFR expression. A large number of clinical trial studies (7) have shown that small molecule inhibitors targeting EGFR have a good effect on the treatment of NSCLC, so small molecule tyrosine kinase inhibitors (TKIs) are the first-line treatment for patients with locally advanced or metastatic NSCLC with EGFR gene mutations.

The discovery of genetic targets has brought infinite possibilities for the treatment of advanced lung cancer, but cardiovascular complications have forced anticancer therapy to be temporarily or prematurely terminated, reducing quality of life and even leading to premature death. This paper focuses on the cardiotoxic side effects and possible mechanisms associated with TKIs of several common targeted therapy drugs recommended by the Chinese Society of Clinical Oncology (CSCO) in 2020.

## 2 Epidermal growth factor receptor tyrosine kinase inhibitors

EGFR is a member of the ErbB family of intoxicine kinase receptors, which has four closely related members: EGFR/HER-1 (ErbB1), HER-2 (ErbB2), HER-3 (ErbB3), and HER-4 (ErbB4) (8). Since the discovery of anti-EGFR therapy for cancer, a variety of epidermal growth factor receptor tyrosine kinase inhibitors (EGFR-TKIs) have been synthesized and approved for clinical treatment. Resistance to first- and second-generation EGFR-TKIs inhibitors has enabled the discovery and development of third-generation TKIs inhibitors. Recently, fourth-generation EGFR-TKIs inhibitors have been clinically evaluated against the third-generation EGFR mutation (C797S) (9). Although the incidence of cardiotoxicity caused by TKIs is very low, there are increasing reports of cardiotoxicity caused by TKIs, which has gradually become the focus of cardiovascular and oncology scientists. The direct effects of TKIs on cardiomyocytes can lead to heart failure, cardiomyopathy, conduction alterations, and prolonged QT intervals, sometimes result in malignant arrhythmias and even cardiac arrest. In addition to cardiac effects, TKIs can also raise vascular effects, leading to arterial hypertension, arterial injury, and venous thromboembolism (10). Cardiotoxicity is one of the most challenging side effects of TKIs, and each TKIs may act on the cardiovascular system in a variety of ways through different targets (see **Table 1**).

**Table 1:** Cardiotoxicity associated with EGFR-TKIs.

EGFR-TKIs		Related cardiotoxicity		Targets
		Instructions	Related literature	
1st Generation	Gefitinib	No relevant description	Acute coronary syndrome, cardiomyopathy, prolonged QT interval	Wild type and certain mutant EGFR
	Erlotinib	Myocardial infarction or ischemia, arrhythmia	Myocardial infarction, ischemia, prolonged QT interval, and cardiomyopathy	HER-1 /EGFR
	Icotinib	No relevant description	No relevant description	EGFR 19 deletion or L858R mutation
2nd Generation	Afatinib	Left ventricular dysfunction	Left ventricular dysfunction	The cysteine 797 site of EGFR and the corresponding cysteine 805 and 803 sites in HER-2 and HER-4
	Dacomtinib	No relevant description	No relevant description	EGFR/HER-1, HHER-2 and HER-4
3rd Generation	Osimertinib	Prolonged QT interval, asymptomatic decrease in left ventricular ejection fraction, symptomatic congestive heart failure	Heart failure, prolonged QT interval, pericardial effusion, myocarditis, and atrial fibrillation	EGFR and HER-2 with T790M mutation

### 2.1 First-generation EGFR-TKIs

#### 2.1.1 Gefitinib

Gefitinib (GEF) acts reversibly on wild-type and certain mutant EGFRs and inhibits the autologous phosphorylation of EGFR tyrosine, thereby further inhibiting downstream signaling and promoting tumor metastasis. Unlike other tyrosine kinase inhibitors, GEF is not considered to be cardiotoxic and is not described in the label, but there has been an increase in reports of cardiotoxicity associated with GEF in recent years.

Lynch et al. (11) reported a case of GEF-related recurrent myocardial infarction. Other literature (12) has reported that the most likely cardiotoxicity in GEF therapy is acute coronary syndrome, and it is speculated that the risk is due to increased platelet reactivity. The first hypothesis is that GEF significantly increases the ability of platelets to produce thromboxane A2, thereby promoting thrombosis. Another hypothesis is that GEF directly damages the atherosclerotic plaque, causing it to rupture.

Notably, Shizuoka Cancer Center in Japan reported (13) a 56-year-old woman diagnosed with GEF-induced cardiomyopathy 7 months after GEF treatment for advanced NSCLC; Symptoms gradually improve after discontinuation of GEF and administration of angiotensin-converting enzyme inhibitors and B-blockers; After 3 months, there was a marked improvement in left ventricular ejection fraction (58% versus 28%), and chest x-ray showed improvement in cardiac enlargement. These results suggest that cardiomyopathy is reversible after discontinuation of the causative drug. This suggests that GEF may be at potential risk of cardiomyopathy. Therefore, when there are unexplained clinical symptoms of cardiomyopathy in patients, it is recommended to complete cardiac magnetic resonance imaging and myocardial biopsy to further determine whether it is caused by cardiac causes, but the specific mechanism of cardiomyopathy caused by GEF has not been clearly reported. It has also been reported in the literature (14, 15) that GEF may prolong the QT interval, but its reliability and mechanism are unknown due to the lack of clinical and experimental data.

In summary, the main cardiotoxic reactions that may be caused by GEF are acute coronary syndrome, cardiomyopathy, and QT interval prolongation. Alhoshani et al. (16) proposed that GEF induces cardiotoxicity by regulating the expression/function of the cardiac PTEN/Akt/Fox03a pathway and the formation of CYP1A1-induced reactive metabolites due to *in vivo* and *in vitro* rat studies, but due to the limited available information on the mechanism of cardiotoxicity caused by GEF, the exact mechanism needs to be further studied.

#### 2.1.2 Erlotinib

Erlotinib is a potent and selective EGFR TKIs that blocks tumor cell division in EGFR-overexpressing human tumor cells, produces cell cycle arrest, and initiates programmed cell death which will inhibit the binding of ATP to the EGFR intracellular tyrosine kinase domain, thereby inhibiting receptor intracellular phosphorylation and blocking downstream signal transduction. Erlotinib drug inserts and the U.S. Food and Drug Administration (FDA) mention cardiovascular complications including myocardial ischemia or infarction and cardiac rhythm failure. In addition, it has been reported in the literature that patients taking erlotinib have a prolonged QT interval after treatment initiation (median QTc prolongation ranges from 7~24 ms) (17). Pinquie et al. (18) also reported a new case of dilated cardiomyopathy associated with erlotinib therapy, suggesting that long-term maintenance therapy with erlotinib may cause dilated cardiomyopathy. Kenji et al. (19) report a case of cardiomyopathy that developed during erlotinib treatment for NSCLC. Two months after erlotinib initiation, our 70-year-old female patient complained of progressive dyspnea, and a diagnostic endomyocardial biopsy confirmed non-specific cardiomyopathy, indicating erlotinib-induced cardiomyopathy.

How erlotinib causes cardiotoxic side effects has not yet been reliably documented, but it has been suggested in the literature (20) that erlotinib may be associated with a lower risk of cardiotoxicity, not necessarily because it inhibits EGFR alone, but because the signal sensor and activator of transcription 3 signaling are upregulated, allowing adaptive fatty acid metabolism to maintain cardiac function, so the specific mechanism of cardiovascular complications caused by targeted drugs may need to be comprehensively analyzed.

#### 2.1.3 Icotinib

Icotinib is a first-generation EGFR-TKIs approved by the National Medical Products Administration of China (NMPA) and is currently only available in China for the treatment of EGFR 19-deletion or L858R-mutated NSCLC, with a similar molecular structure to two first-generation EGFR-TKIs (GEF and erlotinib) (21). Compared with GEF, icotinib has similar efficacy but a better safety profile (22). Although there are increasing literature reports of cardiotoxicity caused by GEF, icotinib, which is similar in molecular structure, has not been described in the drug label or in the literature.

It is worth noting that Peng et al. (23) reported that icotinib can significantly reduce the right ventricular systolic blood pressure and right ventricular hypertrophy index of cytroline-induced pulmonary hypertension in rats, and improve the pulmonary vascular remodeling induced by monocrotaline, and proposed that this effect may prevent the dysfunction of pulmonary artery smooth muscle cells by inhibiting the EGFR-Akt/ERK signaling pathway.

### 2.2 Second-generation EGFR-TKIS

#### 2.2.1 Afatinib

Afatinib is a selective inhibitor of the ErbB family receptor slightly cohort kinase, irreversibly binding to cysteine 797 in EGFR and the corresponding cysteine 805 and 803 in HER-2 and HER4 (24).

Nuvola et al. (25) reported a 71-year-old female smoker with stage 4 EGFR-mutant lung cancer with a previous diagnosis of atrial fibrillation and hypertension, with a left ventricular ejection fraction of 60% on baseline echocardiography, 40% left ventricular ejection fraction, diastolic dysfunction, left ventricular dilation, and pericardial effusion on echocardiography 1 month after treatment with afratinib, and normal left ventricular ejection fraction (60%) 1 week after discontinuation of afatinib, thus presumed that cardiac dysfunction was related to HER-2 inhibition.

#### 2.2.2 Dacomtinib

Dactinib irreversibly inhibits EGFR and is the first-line treatment for patients with EGFR mutation-positive advanced lung cancer, with activity against all 3 kinase-active ErbB family members (EGFR/HER-1, HER-2, and HER-4). Dactinib is superior to GEF in terms of progression-free survival and duration of response (26).

Trials have shown that dacrotinib treatment lacks clinically relevant evidence on the effects of QT interval, heart rate, or PR interval (27), and no cardiotoxicity has been described in the drug label or other relevant literature reports.

### 2.3 Third-generation EGFR-TKIS

Osimertinib is a third-generation irreversible tyrosine kinase inhibitor approved by the European Medicines Agency for patients with EGFR mutations with the T790M mutation (28). Osimertinib was shown to have a favorable safety profile compared to first-generation EGFR-TKIs, with a lower incidence of adverse event grade = 3 (42% vs 47%), however, the incidence of cardiotoxicity was increased in the osimertinib-treated group, and an analysis of the FDA’s adverse events database found that osimertinib raised the incidence of atrial fibrillation, ECG QT interval prolongation, and heart failure compared with first- or second-generation EGFR-TKIs (29). Kartik et al. reported that the reporting odds ratio (ROR) for cardiac failure, AF, and QT prolongation were higher due to the treatment of osimertinib compared with other TKIs. Electrocardiographic monitoring for QT prolongation and monitoring for signs and symptoms of heart failure should be considered in patients taking osimertinib (30). Karishma et al. presented a case of acute, severe biventricular cardio- myopathy due to osimeritinib in a patient with metastatic lung adenocarcinoma and malignant pericardial tamponade (31).

Osimertinib has been reported to cause reversible heart failure in cases (32–36), and 21 of the 558 patients treated with osimertinib in two trials conducted by Piper-Vallillo et al. (37) developed heart failure. It has been speculated that osimertinib-induced heart failure may be related to its inhibition of HER-2 (38). In the case shared by Schiefer et al. (39), a patient suddenly developed subgrade QT interval prolongation (560 ms) after 11 months of treatment with osimertinib, and the QT interval returned to normal within 5 days of drug withdrawal. Osimertinib has also been reported to cause severe cardiac dysfunction, such as myocarditis. Oyakawa et al. (40) reported a case of myocarditis caused by osimertinib, which showed no improvement in left ventricular ejection fraction 12 weeks after discontinuation of osimertinib. In summary, the cardiotoxicity that osimertinib may cause may not be limited to a few described conditions in the label, and the mechanism of cardiotoxicity imposed by osimertinib is not yet known, so caution is required during treatment. Osimertinib (41) may lead to Takotsubo (stress) cardiomyopathy (TC), which has the possibility of cause of heart failure, and osimertinib should not be resumed in patients diagnosed with symptomatic heart failure due to TC induced by osimertinib.

Numerous reports of cardiotoxicity after treatment with TKIs have exposed gaps in the prediction of cardiotoxic side effects from current preclinical drug trials. The diversity of cardiovascular complications caused by TKIs, the older age of most patients with NSCLC, and the fact that most patients have comorbid cardiovascular disease make cardiac management of patients with NSCLC more difficult. Unfortunately, there is currently limited understanding of the mechanisms underlying cardiotoxicity caused by TKIs, and there is no reliable way to predict cardiotoxicity during the treatment of TKIs. Therefore, cardiovascular surveillance for patients with advanced lung cancer receiving targeted drug therapy should not be underestimated. In order to strike for a balance between “life-saving” and “heart-to-heart”, it is essential to implement preventive measures to identify patients at risk of cardiotoxicity throughout the course of targeted drug therapy.

Liraglutin, a glucagon-like brain 1 receptor agonist, has a strong cardioprotective effect, the mechanism of which is not well understood. There has been experimental evidence abroad showing that Liralu protects the heart from GEF-induced cardiac damage through its antioxidant properties and activation of survival kinase (42). The mechanism may provide protection for Liraluf by upregulating survival kinases (ERK1/2 and Akt) and downregulating stress-activated kinases (JNK and P38). There is no reliable clinical data on whether the use of lirarum can actually avoid the cardiac damage caused by GEF, and more animal experiments are needed to verify this idea.

HER-2 is a member of the transmembrane receptor family of tyrosine kinases, expressed in cardiomyocyte membranes, and plays a role in cardiomyocyte growth, survival, and protection against cardiotoxins. As a representative drug of HER-2 inhibitors, the mechanism of cardiotoxic side effects of trastuzumab is believed to be related to its inhibition of HER-2, and whether the mechanism of cardiovascular toxicity caused by TKIs such as afatinib, dactinib and osimertinib, which also inhibit HER-2, is similar to that of trastuzumab needs to be verified by more experimental data.

## 3 Evaluation and monitoring of EGFR-TKIs-induced cardiotoxicity

Although it is not possible to accurately predict the risk factors for the development of cardiotoxicity in patients treated with EGFR-TKIs, patient-specific complications should be considered in the drug selection process to understand the cardiotoxicity of each EGFR-TKIs. Clinical physicians need to balance the risk-benefit ratio between targeted therapy for malignant tumors and drug-induced cardiac toxicity. Therefore, EGFR-TKIs-induced cardiotoxicity safety profile, baseline risk assessment, active surveillance, and prophylactic treatment should be included as part of clinical work (43). The range of cardiotoxicity induced by EGFR-TKIs varies with the specific drug and is influenced by underlying cardiovascular disease or risk factors. Before initiating treatment with EGFR-TKIs, a rigorous baseline risk assessment must be performed on all patients, and baseline cardiovascular risk factors, including obesity, diabetes, hypertension, smoking, etc., must be carefully considered. Cardiac assessment at baseline level, regular dynamic monitoring in treatment with EGFR-TKIs, and post-treatment follow-up, including blood pressure measurement and electrocardiogram, cardiac imaging, and dynamic monitoring of biomarkers, should be routinely performed to determine whether patients would benefit from treatment with EGFR-TKIs and to adjust treatment prior to irreversible cardiac injury (44).

### 3.1 The role of ECG in the assessment and monitoring of EGFR-TKIs-induced cardiotoxicity

ECG can be used to detect some signs of cardiovascular toxicity, such as increased heart rate at rest, ST-T changes, conduction system abnormalities, QT interval prolongation, or arrhythmias. However, ECG changes are often nonspecific and are often influenced by many factors. ECG changes are sometimes transient and unrelated to the progression of chronic heart disease.

### 3.2 The role of cardiac imaging in the assessment and monitoring of TKIs-induced cardiotoxicity

Cardiac imaging includes echocardiography, nuclear imaging, and magnetic resonance imaging (MRI), which can be used for early detection of cardiac toxicity. The purpose of cardiac imaging is to assess the structure and function of the heart and to identify early heart damage. Echocardiography is a non-invasive tool for measuring cardiac function without radiation exposure, and as a result, is still widely used. Compared to two-dimensional (2D) echocardiography, three-dimensional(3D) echocardiography and cardiac magnetic resonance imaging (CMR) provide quantitative volume analysis with greater accuracy and reproducibility (45). LVEF is the most commonly used indicator of mental dysfunction. Current definition of cancer therapeutics-related cardiac dysfunction (CTRCD) is a >10% decrease in LVEF from the previous level and below the lower limit of 50% of normal (46). In addition, compared with two-dimensional echocardiography, three-dimensional echocardiography has higher repeatability, and the measured LVEF has a good correlation with the LVEF measured by cardiac magnetic resonance, which is considered to be the preferred technique for monitoring cardiac insufficiency and cardiovascular toxicity in cancer patients. However, LVEF changes occur only after substantial myocardial injury and decompensation, and variability can be as high as 10% when measured, resulting in low LVEF sensitivity and difficulty in detecting subclinical myocardial injury (47, 48). The application of 2D spot tracking technology and ultrasound strain analysis can detect early myocardial injury. Strain echocardiography is a measure of morpho structural changes in the heart muscle that can provide a global and local assessment of cardiac function. Current studies in antineoplastic treatment of cardiac impairment have demonstrated that GLS assesses left ventricular systolic function more sensitively than LVEF, and a 15% decrease in GLS from baseline is suggestive of early subclinical left ventricular dysfunction. Left ventricular GLS has been recognized as the most sensitive indicator for early monitoring of cardiotoxicity by the American Society of Echocardiography (ASE), the European Society of Cardiovascular Imaging (EACVI), and the European Association of Heart Diseases (ESC) (49). Multi-layer radionuclide angiography was used to evaluate the cardiovascular toxicity and left ventricular function induced by targeted drug therapy with good accuracy and reproducibility, and there were few technical limitations. However, cardiac nuclear imaging is not commonly used to monitor cardiotoxicity because it provides only limited information about cardiac structure and hemodynamics and is limited by radiation exposure (50). When the time and possibility of reversibility of cardiac insufficiency caused by TKIs are not clear, CMR can be used as an important assessment tool to identify, and diagnose early cardiac damage that may be caused by such drugs by performing baseline and periodic cardiac vascular assessments in patients receiving targeted drug therapy (51). One study (52) showed that CMR assessed a decrease in LVEF and GLS in patients treated with low-dose anthracyclines from baseline to after 6 months. CMR can assess cardiac structure and function, measure left ventricular chamber size and systolic function, and provide quantification of chamber size and LVEF, independent of geometric assumptions and acoustic windows. In conclusion, CMR is preferred over echocardiography when more reliable LVEF measurements and assessment of early cardiac damage are required (53).

### 3.3 Role of biomarkers in the assessment and monitoring of TKIs-induced cardiotoxicity

As an important tool for the diagnosis of cardiovascular diseases, biomarkers have become increasingly valuable in the baseline risk assessment and diagnosis of myocardial injury in cancer patients in recent years. Studies (54) have shown that multi-targeted tyrosine kinase growth and angiogenesis inhibitors exert cardiotoxic effects by inhibiting vascular endothelial growth factor and vascular endothelial growth factor receptor tyrosine kinases from damaging vascular endothelial cells and disrupting cardiac contractility and vasodilation. Therefore, by identifying potential biomarkers that predict cardiotoxicity, it is expected that early detection of cardiotoxicity can guide treatment and improve the prognosis of patients on anticancer therapy.

#### 3.3.1 The role of cardiac troponin in the assessment and monitoring of cardiotoxicity induced by EGFR-TKIs

In anticancer therapy, cancer itself and cardiotoxicity caused by anticancer therapy can also trigger abnormal expression of cTn through cell damage, oxidative stress, fibrosis and other pathways. A recent meta-analysis (55) showed that anticancer therapy can lead to an increase in serum cTn levels, and that increased cTn is associated with systolic dysfunction in patients receiving anticancer therapy, suggesting that it is of great value in predicting left ventricular dysfunction and warrants further investigation. Another study on the cardiotoxicity of anthracyclines in breast cancer patients (56) also showed that anthracyclines can increase hs-cTnT levels, and that an increase in hs-cTnT levels at the end of anthracycline therapy may indicate subsequent cardiotoxicity. In recent years, cTn has been widely used in clinical practice due to its advantages of simple detection, low cost, and high diagnostic value. Routine monitoring of cTn for early detection of cardiotoxicity in patients receiving anticancer therapy has become a trend. The latest European Society of Medical Oncology (ESMO) consensus also recommends that both baseline measurement and regular monitoring of hs-cTnTI/T be considered in high-risk patients (with prior cardiovascular disease) and those receiving high-dose cardiotoxic chemotherapy (e.g., anthracyclines) (57).

#### 3.3.2 Brain natriuretic peptide and N-terminal prohormone of brain natriuretic peptide in the assessment and monitoring of TKIs-induced cardiotoxicity

BNP and NT-proBNP are hormones secreted under the stimulation of factors such as cardiomyocyte stretching, neurohormone activation and myocardial hypoxia, which can act on distant tissues and have the effects of diuresis, vasodilation and regulation of the body’s water and sodium balance, both of which are widely used as clinical markers of heart failure and can also be applied in the monitoring of chemotherapy-induced left ventricular dysfunction. Studies [35] have shown that there is a consistent temporal correlation between NT-proBNP and cardiotoxicity during long-term follow-up, suggesting that NT-proBNP has some significance in predicting cardiotoxicity. BNP and NT-proBNP also have certain limitations as cardiotoxicity biomarkers, and their predictive and diagnostic value for cardiotoxicity caused by TKIs still needs to be further studied and verified. Due to its low cost and ease of use, BNP/NT-proBNP can be selected as a biomarker of cardiotoxicity in patients treated with cancer for cardiac function monitoring.

#### 3.3.3 Soluble suppression of tumorigenecity-2, sST2

Role in the assessment and monitoring of EGFR-TKIs-induced cardiotoxicity sST2 as a biomarker of inflammation, fibrosis, and myocardial stress in the diagnosis and prognosis of heart failure and myocardial infarction has been increasingly studied. Studies (58) have shown that sST2 is less affected by age than NT-proBNP or hs-TnT, contributing to more accurate risk stratification and prognostic management of heart failure. A previous study (59) showed an increase in sST2 levels in breast cancer patients during and after anthracycline therapy, however, the study did not specify whether the elevated sST2 levels were caused by the breast cancer itself or by anthracyclines. Another study (60) followed breast cancer patients who received radiotherapy and found that sST2 levels were inversely correlated with cardiac systolic function. These studies suggest the potential value of sST2 in monitoring anticancer therapy-related dysfunction and its prognosis, and that larger clinical sample sizes, longer follow-up periods, and better clinical trial design are needed in the future to validate the effectiveness of sST2.

## 4 Conclusion

In summary, the innovative development of new anti-tumor drugs has significantly improved the overall survival rate of patients with malignant tumors, but also produced more adverse reactions. In addition to secondary malignancies, the life-threatening complication of the treatment of malignant tumors is the induction of cardiotoxicity by targeted drugs. Therefore, a multidisciplinary approach is essential to find a balance between the need for targeted therapy of EGFR-TKIs and the potential induction of cardiotoxicity in the use of EGFR-TKIs. As with other diseases, prevention is better than cure, and in the course of targeted therapy for EGFR-TKIs, it is important to understand the cardiotoxicity induced by TKIs, according to our review of the literature. The cardiotoxic effects of EGFR-TKIs works diversely, and although there are commonalities, the main adverse effects are different among the drugs in question. This means that cardiotoxicity is not caused by a single mechanism of action of EGFR-TKIs, but varies from drug to drug. Secondly, each patient on EGFR-TKIs-targeted therapy should be carefully evaluated and monitored, including ECG, cardiac imaging, and biomarkers, to address susceptibility risk factors as early as possible and to appropriate treatment or drug modification for emerging cardiotoxicity. In conclusion, the clinically relevant results of EGFR-TKIs are combined with genetic, imaging features and biomarkers to evaluate EGFR-TKIs-targeted therapy at the baseline level and provide effective data support, so that more patients with malignancies can benefit from it.

## Author contributions

YW: Conceptualization, Data curation, Investigation, Methodology, Writing – review & editing. QQ: Data curation, Methodology, Writing – review & editing, Resources. XD: Data curation, Methodology, Writing – review & editing, Investigation. MW: Conceptualization, Funding acquisition, Project administration, Writing – original draft.
